# Supplementary material for: Single-Cell Transcriptomics Reveals the Complexity of the Tumor Microenvironment of Treatment-Naive Osteosarcoma
Source: Front Oncol. 2021 Jul 21;11:709210. doi: 10.3389/fonc.2021.709210 (PMC8335545; doi:10.3389/fonc.2021.709210)
Supplement: Supplementary Document 1 — Kaplan-Meier curve of the 518 genes associated with survival. [file DataSheet_1.zip › Supplementary Document 1/sur.SYT12.pdf]

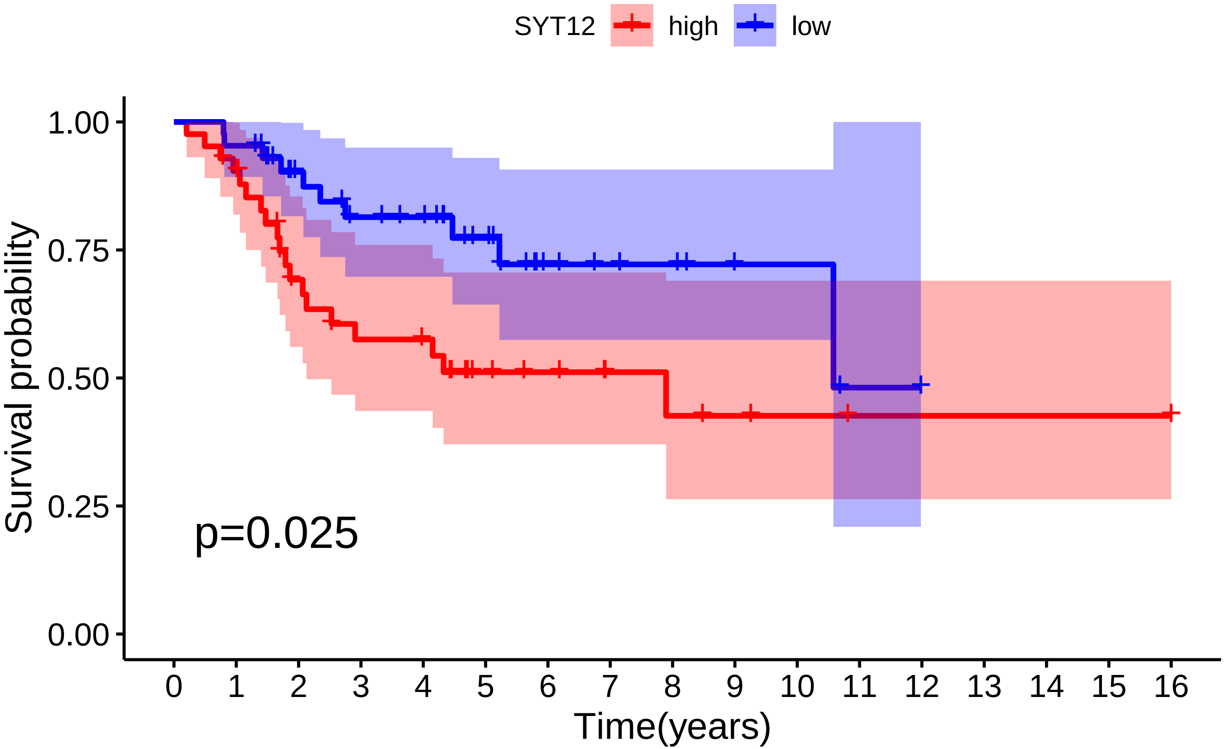


| SYT12 | 0 | 1 | 2 | 3 | 4 | 5 | 6 | 7 | 8 | 9 | 10 | 11 | 12 | 13 | 14 | 15 | 16 |
|---|---|---|---|---|---|---|---|---|---|---|---|---|---|---|---|---|---|
| high | 42 | 37 | 24 | 19 | 18 | 11 | 9 | 6 | 5 | 4 | 2 | 1 | 1 | 1 | 1 | 1 | 1 |
| low | 43 | 41 | 31 | 26 | 24 | 17 | 9 | 7 | 6 | 3 | 3 | 1 | 0 | 0 | 0 | 0 | 0 |

Time(years)
